# The occurrence of a particular state is a predictor of successful travel consultation

**DOI:** 10.1371/journal.pone.0352101

**Published:** 2026-06-22

**Authors:** Hidehito Honda, Takatomi Kubo, Ryosuke Hisamatsu, Yoshimasa Ohmoto, Kazushi Ikeda, Kazuhiro Ueda

**Affiliations:** 1 Graduate School of Arts and Sciences, The University of Tokyo, Meguro-ku, Tokyo, Japan; 2 Graduate School of Science and Technology, Nara Institute of Science and Technology, Ikoma-shi, Nara, Japan; 3 Graduate School of Interdisciplinary Information Studies, The University of Tokyo, Bunkyo-ku, Tokyo, Japan; 4 Faculty of Informatics, Shizuoka University, Hamamatsu-shi, Shizuoka, Japan; IESEG School of Management, FRANCE

## Abstract

Dyadic interactions, such as consultations between clerks (i.e., travel agent sales staff) and customers, involve multiple behaviors that reflect people’s internal states, such as interests and preferences. Thus, the time-series patterns of multiple behaviors between two people reveal the characteristics of the interaction. In this study, we conducted an experiment on travel consultations between clerks and customers, which was as close as possible to a real situation, and analyzed the time-series shifts of multiple behaviors in a 30-minute dyadic interaction using a hidden Markov model. We found differences in interactions between successful and unsuccessful consultations. In particular, in the successful interaction, a customer entered an interactional state associated with interest in the content of the consultation in the first 10 minutes, and such a state was likely to persist during the consultation. These findings suggest that early interactional engagement may serve as a potential behavioral marker associated with consultation success and offer a preliminary process-level perspective on interaction dynamics in naturalistic dyadic consultations.

## Introduction

Inferences about people’s intentions, emotions, and behaviors are highly important for a smooth interaction [1–6]. Previous studies have shown that human behaviors, such as gaze, posture, prosody, and head position, are correlated with people’s internal states (e.g., interest, persuasiveness, and satisfaction) [7–12]. Thus, each behavior not only is correlated with the internal state but may also play an important role in achieving smooth communication in person-to-person interactions such that the manifestation of a certain behavior can be a signal for inferring people’s internal states. Each behavior is expected not to appear independently, but in a dependent and composite manner. Furthermore, there may be a time-series pattern in the manifestation of behaviors. In other words, in human communication, the interactive processes of multiple behaviors between people are closely related to smooth communication [13–19].

In this study, we examined interactions in dyadic human-to-human communication during a 30-minute travel consultation. In the travel consultation, a customer tells a clerk (i.e., travel agent sales staff) where they want to go, stay, go sightseeing, and many other things. Based on these requests, the clerk proposes a travel plan. In this process, interactions are expected to appear; the customer may react to the clerk’s suggestions, and the clerk may infer whether the customer is interested in the proposed plan according to the customer’s reactions. Previous studies examining consultation interactions, such as those conducted in psychotherapy contexts [20–22], are relevant to the present research. We believe that the analysis of interactions in different consultation situations can provide useful insights into both new situations (i.e., context-specific insights) and, more broadly, the nature of human interaction (i.e., general insights).

Here, we highlight the important factors that affect interactions during a 30-minute consultation. For example, after participating in a 30-minute travel consultation, you may think that “the consultation was very informative.” In other cases, you may think, “The consultation was not informative. I could not obtain good information.” This is true not only for the customer, but also for the clerk. In some cases, the clerk may feel “I was able to provide good information,” while in other cases, they may feel “I was not able to provide good information.” That is, a 30-minute consultation may be “successful” or “not successful” for both sides. How does this interaction vary when the consultation is successful or unsuccessful? It is well known that after experiencing an event that lasts for a certain amount of time, people make unique evaluations of the event [23–25]. These findings suggest that time-series processes that determine successful or unsuccessful consultations exist during a 30-minute period. Such processes may be reflected in dyadic interactions between clerks and customers. In parallel, research on service interactions has suggested that early phases of an interaction—such as initial rapport formation and customer engagement—may influence subsequent interaction dynamics and overall satisfaction [26,27]. However, most of this research has relied on retrospective or aggregate measures and has not examined how such early-stage processes emerge from moment-to-moment behavioral interactions in naturalistic settings. Based on this perspective, we expected that early interactional states reflecting customer engagement would be associated with consultation success.

The goal of this study was to clarify the nature of interactions in successful consultations from a time-series perspective. Due to little previous research and limited knowledge on the difference between successful and unsuccessful interactions, it was not possible to use a methodology for hypothesis testing. Therefore, we performed exploratory analyses using the hidden Markov model (HMM), examined the unobserved factors (states) that generated certain behavioral patterns, and identified the state that could predict successful interactions. Previous studies have shown that the HMM is a highly useful method to understand human behaviors [28]. In fact, the HMM has been employed in research on interactions, such as group interaction or human-robot interaction [29–37]. Thus, we believe that the HMM-based time-series analysis of interactions is highly suitable for achieving the present goal.

The approach adopted in this study using the HMM is summarized as follows. Because sufficient findings have not been accumulated in previous studies, we analyzed the observed data in an exploratory and data-driven manner. For example, in the HMM, we must determine the number of hidden states in the model. As we did not have a specific hypothesis, we determined it based on statistical measures. We then discussed the observed data based on the estimated parameters of the model and considered the internal state of the interaction underlying the behavioral patterns.

## Methods

### Ethics statement

The protocols of the travel consultation experiment conformed to the Declaration of Helsinki. This study was approved by the Ethics Committee for Research Involving Human Participants at the Graduate School of Arts and Sciences, the University of Tokyo (Approval No. 369). All the participants provided written informed consent to participate in the study.

### Participants

A total of 10 clerks (*M*_*age*_ = 30.20, *SD*_*age*_ = 5.47, all women) and 15 customers (11 women and 4 men, *M*_*age*_ = 50.93, *SD*_*age*_ = 10.47) participated in the experiment. The clerks were actual workers at a travel agency in Japan. We recruited customers who were interested in traveling with their families and wanted to consult about their travel plans.

### Experiment, task, and procedure

The experiment was conducted between May 20 and May 28, 2015. A travel consultation task was performed. In this task, a customer consulted a clerk about a travel plan, and the clerk proposed travel plans using brochures. The clerk was required to use the reservation system during the experiment to check the reservation status and confirm whether the reservation could be made according to the customer’s desired schedule. In other words, the experiment was performed as naturally as possible, except that the maximum consultation time was 30 minutes.

Each customer participated in the consultation task twice (consulting with a different clerk each time) and each clerk participated with three different customers. This task was performed with 30 different pairs. The paired clerks and customers did not meet before the experiment. After the travel consultation, customers were asked to indicate how satisfied they were with the travel consultation on a 7-point scale (1 = not at all satisfied to 7 = very satisfied), and clerks were asked to indicate how well they thought the travel consultation went on a 7-point scale (1 = not at all successful to 7 = very successful). In addition, we asked clerks and customers several other questions related to the travel consultation. However, we did not use them in the present analyses. The details of these questions are provided in the Supplementary Material.

We recorded each travel consultation using two cameras: one for clerks and the other for customers. We checked the recorded movies for the 30 pairs and found that 10 behaviors (4 of which were the clerk’s and 6 of which were the customer’s) were widely observed regardless of the pairs. Thus, we focused on these 10 behaviors in the present analyses. Table 1 summarizes the targeted behaviors. Then, for each video datum, we annotated the 10 targeted behaviors using ELAN (https://archive.mpi.nl/tla/elan). In particular, we input information regarding when each behavior started and ended during the 30-minute consultation. Based on this procedure, we obtained data on behaviors that appeared during the travel consultation. We checked the validity of the annotations and found that they were highly consistent regardless of the annotators (details are provided in the Supplementary Material).

**Table 1:** Ten verbal and nonverbal behaviors that were the targets for analyses.

	Target	Type of behavior
1	Clerk	Lean forward
2	Customer	
3	Clerk	Gaze to brochure
4	Customer	
5	Clerk	Speak
6	Customer	
7	Clerk	Gaze to customer
8	Customer	Gaze to clerk
9	Customer	Chin on the hand
10	Customer	Nod

### Analysis procedure using the HMM

We performed a time-series analysis of the nature of the interactions using the HMM. For the analysis, we used data with a time unit of seconds. The analysis steps are outlined below (for the detailed procedure, please refer to the Supplementary Material).

**Fig 1:**
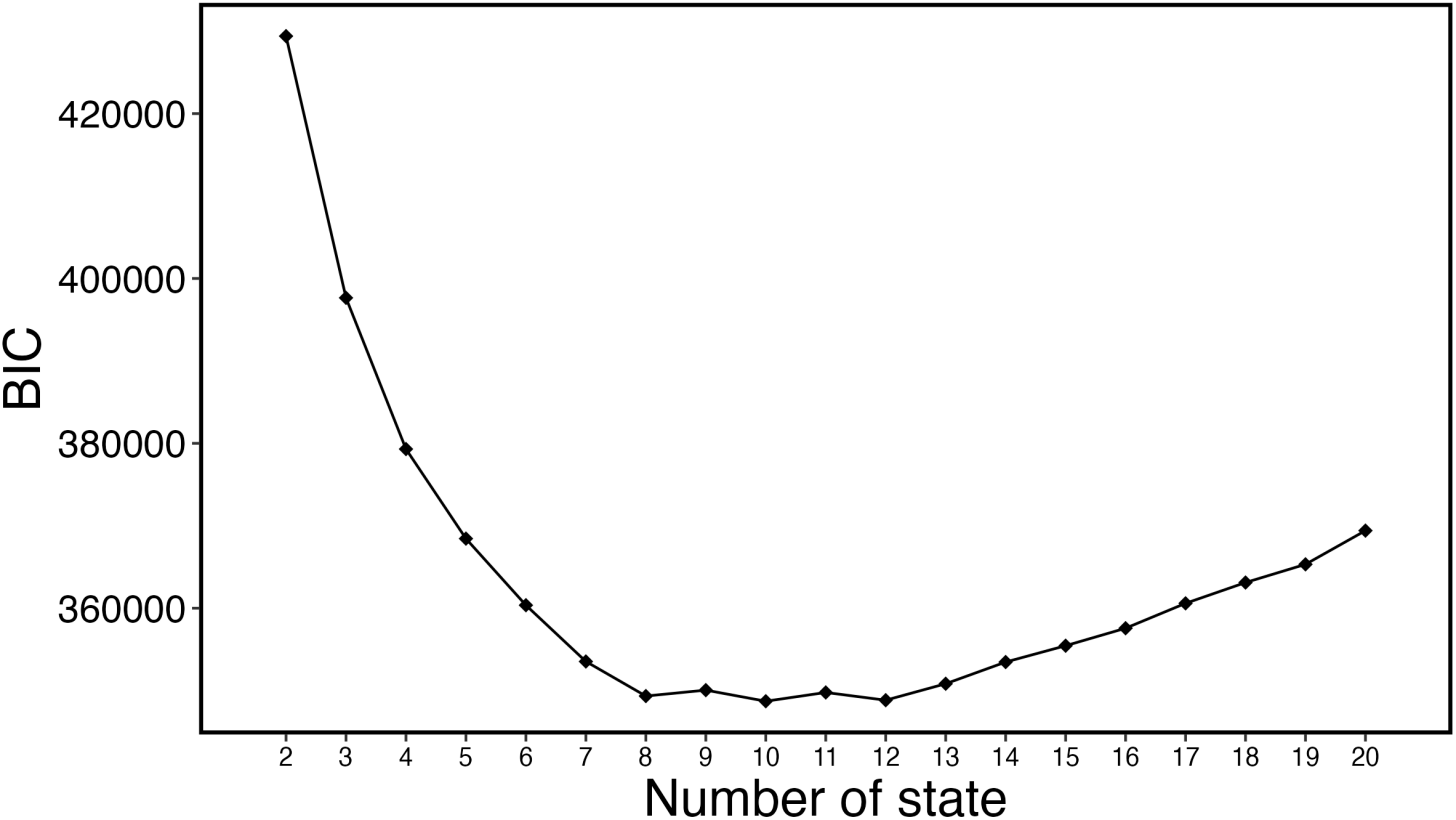
BIC as a function of the number of states.

**Fig 2:**
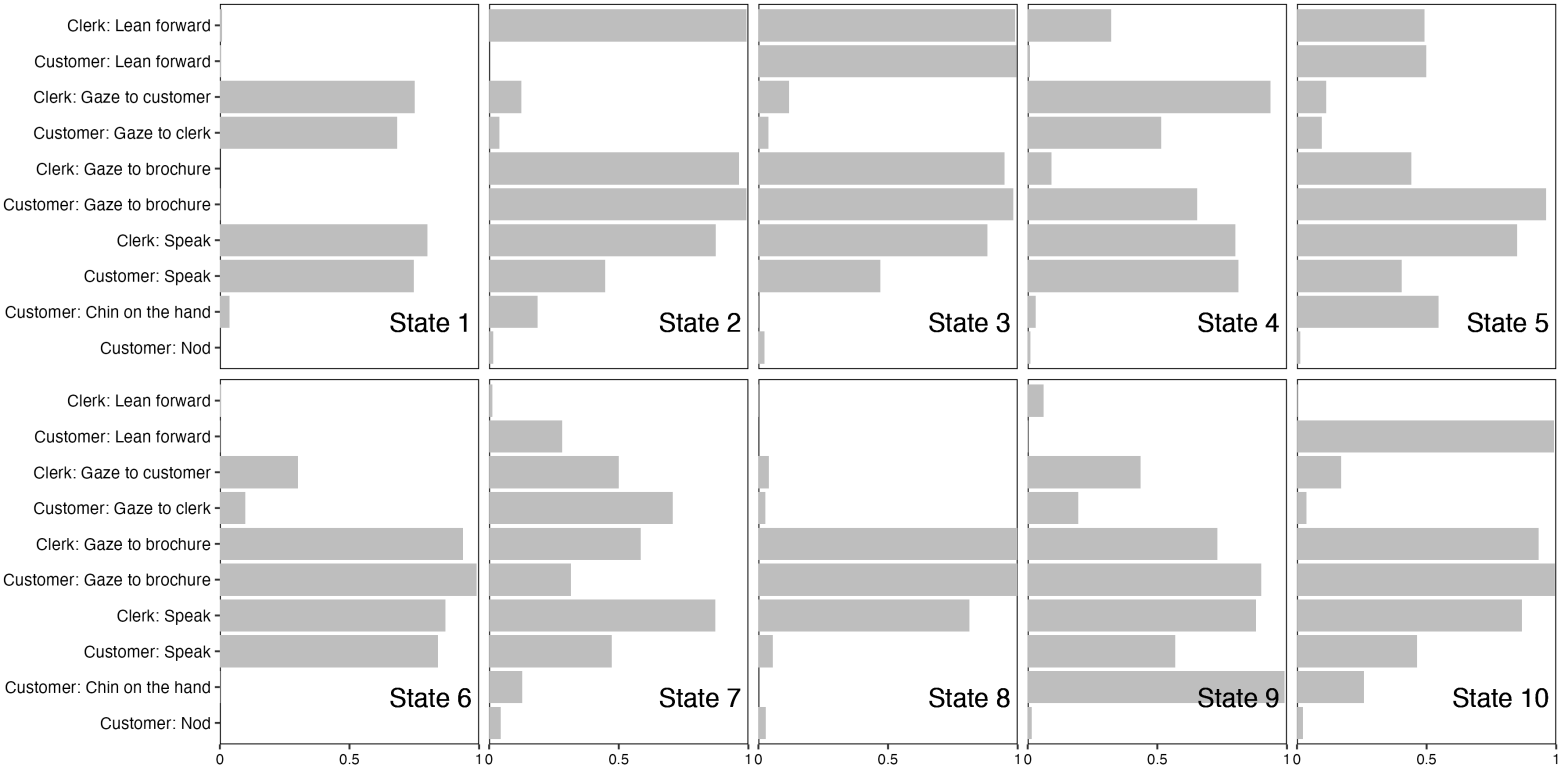
The probability that each state generates each behavior.

1. A certain behavioral pattern appeared every second. See the image of data in Table 2 (a hypothetical example). In this example, in the first second (Time 1), behavioral patterns, such as the clerk’s leaning forward (LF) and speaking and the customer’s leaning forward (LF) and nodding (N), were observed. We examined the behavioral patterns observed at each second, and there were up to 2^10^ (1024) possible behavioral patterns. We used these behavioral patterns for the analyses.
2. We estimated the unobserved (i.e., hidden) states that would generate the observed behavioral patterns. For this estimation, we used the “hmm.discnp” library in R [38].
3. We determined the number of hidden states using BIC in terms of predictability and complexity. As a result, 10 states were selected as the best model (Fig 1), and we employed this model in the analyses.
4. For each hidden state, the emission probability for each behavioral pattern was calculated. Furthermore, based on this probability, we calculated the probability of appearance of each behavior (i.e., the probability that each of the ten behaviors would appear). Fig 2 illustrates this probability. Different behaviors appeared in each state, suggesting that each state represented a different internal state for clerks and customers.
5. We divided each consultation time into 10 segments (i.e., each segment was approximately 3 minutes).
6. For each pair, we calculated the probability of the occurrence of each hidden state in each time segment. That is, for each pair and hidden state, 10 values (i.e., probabilities for 1, 2, …, 9, and 10 time segments) were calculated.
7. To identify the hidden states that would accurately predict a successful consultation (i.e., we tried to categorize the emission patterns of hidden states into “successful” and “unsuccessful” pairs), we performed cluster analyses using the probabilities calculated in the procedure 6. We conducted hierarchical cluster analysis using Ward’s method. Because our goal was to identify the hidden states that would accurately predict a successful consultation, we attempted to find two clusters based on the results of the hierarchical cluster analysis.

**Table 2:** Image of data (a hypothetical example).

	Clerk’s behavior				Customer’s behavior					
Time	LF	G2Cu	G2B	S	LF	G2Cl	G2B	S	C	N
1	1	0	0	1	1	0	0	0	0	1
2	1	0	0	0	1	1	1	0	0	1
3	0	0	1	1	1	1	1	0	1	1
…	…	…	…	…	…	…	…	…	…	…

Note. Abbreviations: (LF: lean forward), (G2Cu: gaze to customer), (G2B: gaze to brochure), (S: speak), (G2Cl: gaze to clerk), (C: chin on the hand), (N: nod). “1” (or “0”) indicates appearance (or non-appearance) of behaviors.

Our method for predicting successful pairs can be summarized as follows. When a particular occurrence pattern in a certain hidden state, which was identified by the result of the cluster analysis, was observed only in successful pairs, we regarded the occurrence pattern of the hidden state as a good predictor of a successful pair. We operationally defined successful travel consultations using the results of the questionnaire for clerks and customers. We defined the consultation as “successful” when both the clerk’s rating was 5 or higher and the customer’s rating was 6 or higher (the distribution of rating is presented in the Supplementary Material). Otherwise, the consultation was operationally defined as “unsuccessful.” In other words, a pair was defined as successful when both the clerk and customer responded that they were satisfied with the travel consultation.

## Results

### Nature of hidden states and prediction of successful consultations

**Fig 3:**
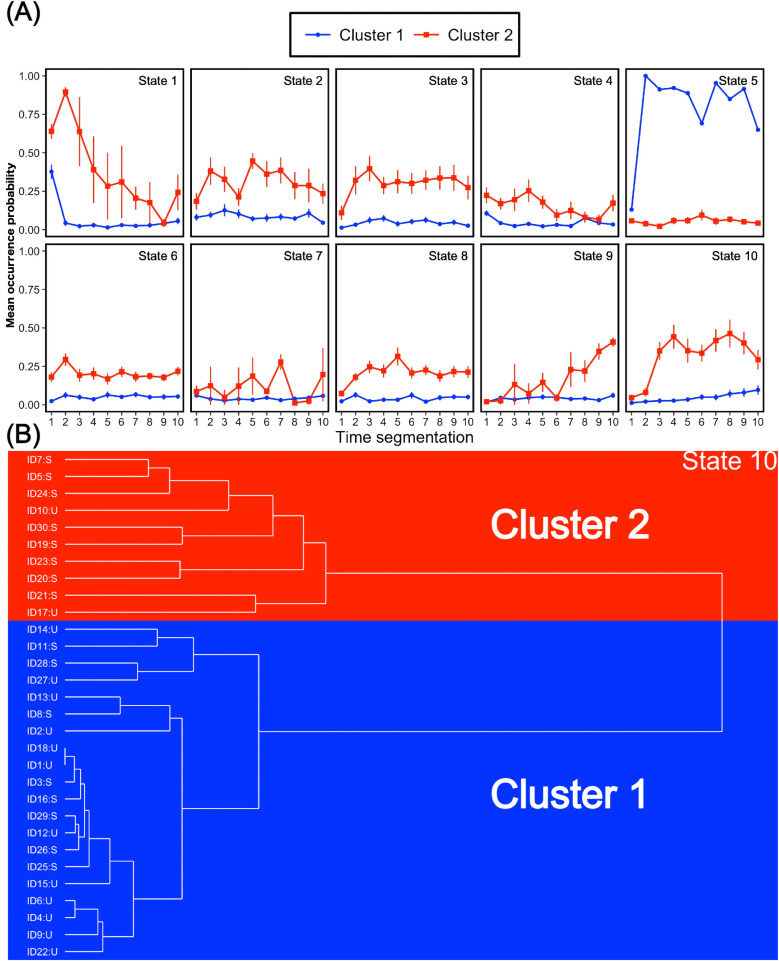
Results of cluster analysis. **(A)** Mean occurrence probabilities for two clusters as a function of time division. The error bar denotes standard error. **(B)** An example of cluster analysis. This figure shows the result for state 10. “S” indicates a successful pair, while “U” indicates an unsuccessful pair.

**Fig 4:**
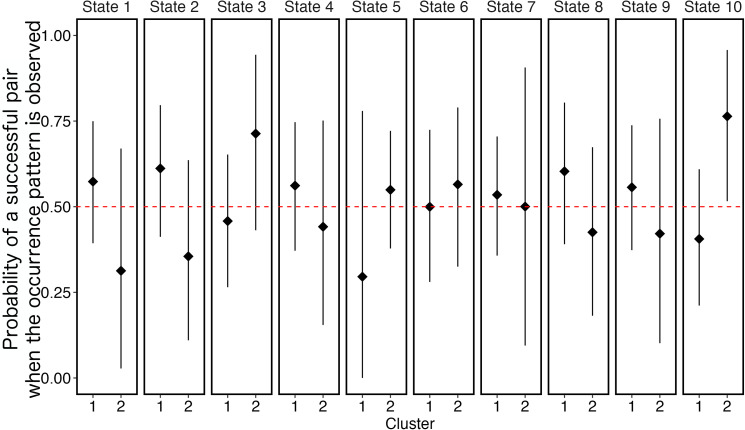
Posterior probability of successful pair when the occurrence pattern is observed. The point denotes the median of posterior distribution. The error bar denotes its 95% highest density interval.

Fig 3(A) shows the mean occurrence probabilities of the two clusters as functions of time segmentation. We then examined which occurrence patterns of the hidden states and clusters could accurately predict successful pairs. For this examination, we calculated the posterior probability of successful pairs when an occurrence pattern defined by cluster 1 or 2 in a certain hidden state was observed. According to the general method [39], we conducted the following analysis. Since we did not have any prior knowledge about the features of successful pairs, we assumed a vague prior distribution using a beta distribution. The beta distribution has two parameters, α and β. When *X* follows a beta distribution, its density *p*(*X*) can be described as


$$\begin{array}{c} p(X)=\frac{\Gamma (\alpha +\beta )}{\Gamma (\alpha )\Gamma (\beta )}X^{\alpha -\text{1}}(\text{1}-X{)}^{\beta -\text{1}}, \end{array}$$ (1)

where Γ(z) is the gamma function. For the vague prior distribution, we set *α* and *β* as 1. Starting with this prior distribution, we computed the posterior distribution after the experiment with *α = 1 + z* and *β =* 1*+* (*N – z*), where *N* indicates the whole number of pairs in each cluster and z indicates the number of successful pairs in the cluster. For example, in state 10, cluster 1 included 20 pairs, of which eight pairs were successful [Fig 3(B)]. In this case, *N* is 20 and *z* is 8 (i.e., *α =* 9 and *β =* 13). We then estimated the probability of successful pairs based on the 95% highest density interval (HDI) for the posterior distribution. Fig 4 shows the posterior probabilities of successful pairs when an occurrence pattern is observed. We found that a travel consultation would succeed with a high probability when the occurrence pattern of cluster 2 in state 10 was observed (median was 76.4%, and 95% HDI was higher than chance level). See cluster 2 in Fig 3 (B). In this cluster, 8 out of 10 pairs were successful.

**Fig 5:**
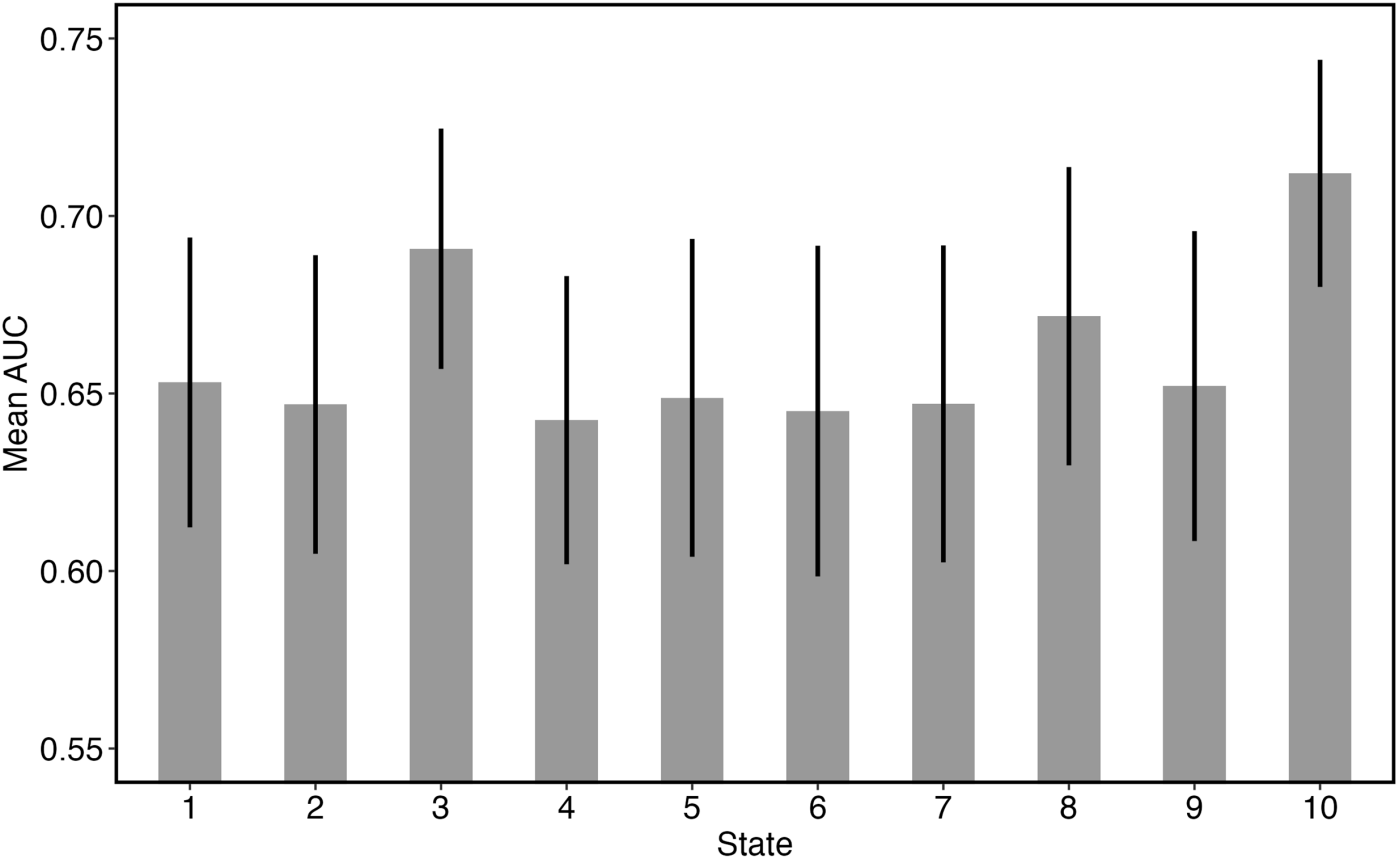
Mean AUC across 200 repeated 10-fold cross-validations (error bars represent ±1 SD).

To further examine whether this relationship generalizes beyond descriptive associations, we conducted a predictive validation analysis using a repeated 10-fold cross-validation procedure [40]. In this analysis, both the training and test sets included observations from the period 6–15 minutes after the consultation began (90 data points = 30 dyads × 3 segments), a period during which State 10 began to emerge prominently among successful dyads. The dataset was randomly partitioned into ten folds, with nine folds used for model training and the remaining fold for testing. This process was repeated 200 times with different random splits, and the area under the ROC curve (AUC) was computed for each iteration. Fig 5 shows the mean AUC values ± standard deviation (SD) across these 200 repetitions. Consistent with the previous Bayesian analysis, the occurrence of State 10 during this period predicted consultation success relatively well (the mean AUC exceeded 0.7), a level of discriminative performance generally considered acceptable and practically informative in applied and behavioral research [41,42]. In contrast, all other states yielded AUC values below 0.7 (results obtained using alternative time windows and segment combinations are reported in the Supplementary Materials).

Here, we discuss the characteristics of state 10. According to the emission probabilities of the 10 behaviors in state 10 (see Fig 2), we can interpret this state as a generating behavior where a clerk and customer speak to each other and watch brochures while the customer is leaning forward. Previous studies indicated that the customer’s behavior of “leaning forward” is highly correlated with the internal state of interest [9,43], suggesting that state 10 represents the customer’s internal state of interest in the clerk’s proposition. Note that cluster 2 of state 10 suddenly appeared in time segmentation 3 (i.e., approximately 6–9 minutes after the start of the whole 30-minute consultation). This suggests that whether the travel consultation was successful or not was shaped early in the consultation.

**Fig 6:**
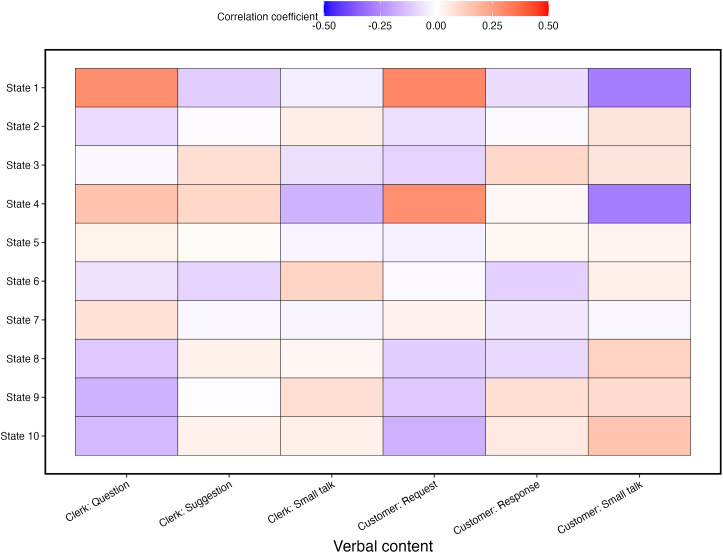
Correlation between hidden-state occurrence probabilities and proportions of verbal content categories.

To further analyze the characteristics of each hidden state, we examined the conversational content between the clerk and the customer. The analytical procedure was as follows. First, among the segments labeled as “Speak,” two individuals with professional experience in travel consultation (i.e., employees working at travel agencies) briefly reviewed the conversational content. Based on their review, it was determined that the utterances could be broadly classified into three categories for both clerks and customers as summarized in Table 3. Following this categorization, two independent coders who were blind to the study hypotheses classified the conversational content of each segment labeled as “Speak.” After the initial coding, the classifications were compared, and any discrepancies were discussed between the two coders until a consensus was reached for the final categorization. We then examined the relationship between the hidden states and the conversational content as follows. For each dyad, we calculated (1) the occurrence probability of each hidden state in each of the ten time segments [as shown in Fig 3(A)] and (2) the proportion of verbal content categories, as defined in Table 3, that occurred within the same time segment. These two variables were computed for all 30 pairs, and we then calculated the correlation coefficients between the occurrence probabilities of the hidden states and the proportions of each verbal content category. The correlation coefficients are presented as a heatmap in Fig 6. As shown in the figure, each hidden state was associated with a distinct pattern of verbal content. In particular, when we focus on State 10, we find that when this state emerged, the clerk’s utterances were less likely to involve “Question,” and the customer’s utterances rarely involved “Request.” Instead, the clerk tended to make “Suggestion.” This pattern is consistent with our interpretation that State 10 reflects a state in which the customer shows interest in the clerk’s suggestion, thereby supporting the validity of the previous discussion.

**Table 3:** Verbal content when behaviors were labeled “Speak”.

	Category	Content
Clerk	Question	Utterances in which the clerk asked open- or closed-ended questions to elicit the customer’s preferences or intentions [e.g., “What are you planning to do at that destination?” (open-ended) or “Would you like accommodation with two meals included per night?” (closed-ended)].
	Suggestion	Utterances in which the clerk proposed specific aspects of the travel plan, such as destinations, means of travel, or product options. This category also included suggestions related to the images or impressions associated with the proposed plan, as well as recommendations made to the customer.
	Small talk	Other verbal content not directly related to the travel consultation.
Customer	Request	Utterances in which the customer expressed specific or abstract requests regarding the travel plan. This category included concrete requests such as “a non-smoking room,” “a plan with two meals,” or “a room with an ocean view,” as well as more abstract or image-based requests such as “I want to relax” or “I want to go sightseeing.” It also included objective explanations using numerical or factual information and descriptive explanations that conveyed impressions or imagery related to the customer’s wishes.
	Response	Response to a question
	Small talk	Other verbal content not directly related to the travel consultation.

### Sensitivity analysis

To assess the robustness of the identified hidden states, we conducted additional sensitivity analyses by varying the random seed and the assumed number of hidden states. The results of the sensitivity analysis are summarized in Table 4.

**Table 4:** Correlation coefficients between the emission probability patterns of the state that achieved the highest AUC in each parameter estimation condition and those of State 10 reported in the main text.

State	Seed	AUC	Correlation coefficient
8	1	0.744	*r* = 0.945, *p* <.01
8	2	0.736	*r* = 0.907, *p* <.01
8	3	0.719	*r* = 0.821, *p* <.01
10	1	0.739	*r* = 0.907, *p* <.01
10	2	0.736	*r* = 0.923, *p* <.01
10	3	0.691	*r* = 0.907, *p* <.01
12	1	0.740	*r* = 0.913, *p* <.01
12	2	0.721	*r* = 0.984, *p* <.01
12	3	0.736	*r* = 0.983, *p* <.01

When the 10-state HMM was re-estimated with three different random seeds, a hidden state with the highest AUC in predicting consultation success consistently emerged. The emission probability profiles of these best-predicting states were strongly correlated with those of State 10 reported above (all r > .90). Similarly, when we fitted 8-state and 12-state HMMs using three random seeds each, we again detected a state with the highest predictive performance whose emission patterns closely resembled those of State 10 (all r > .80).

These results suggest that a state with an emission profile similar to State 10 was consistently recovered across the examined model specifications and random initializations.

### Nature of successful travel consultations

In the previous section, we identified hidden states that predicted successful travel consultations. In particular, in time segmentation 3, state 10 diverged into two occurrence patterns, one of which (cluster 2) predicted a successful travel consultation. In this section, we discuss which transition patterns of hidden states generated the diverging pattern of state 10 in time segmentation 3. For this aim, we examined the transition patterns of hidden states in time segmentation 2 (i.e., immediately before the diverging pattern was observed) for the two clusters with the following procedure. First, we estimated the most probable hidden state in each second for each pair based on the observed behavioral patterns. Next, we calculated the transition probabilities between the hidden states. For each pair, we calculated the transition probabilities in each second [transition patterns were 100 (*s*_*t*_ denoting the state at the targeted time and *s*_*t-1*_ denoting the state 1 second before the targeted time, 10 patterns for *s*_*t*_ × 10 patterns for *s*_*t-1*_)] during the travel consultation. Finally, we calculated the mean transition probability for each cluster (i.e., the mean of 20 pairs for cluster 1 and that of 10 pairs for cluster 2).

**Fig 7:**
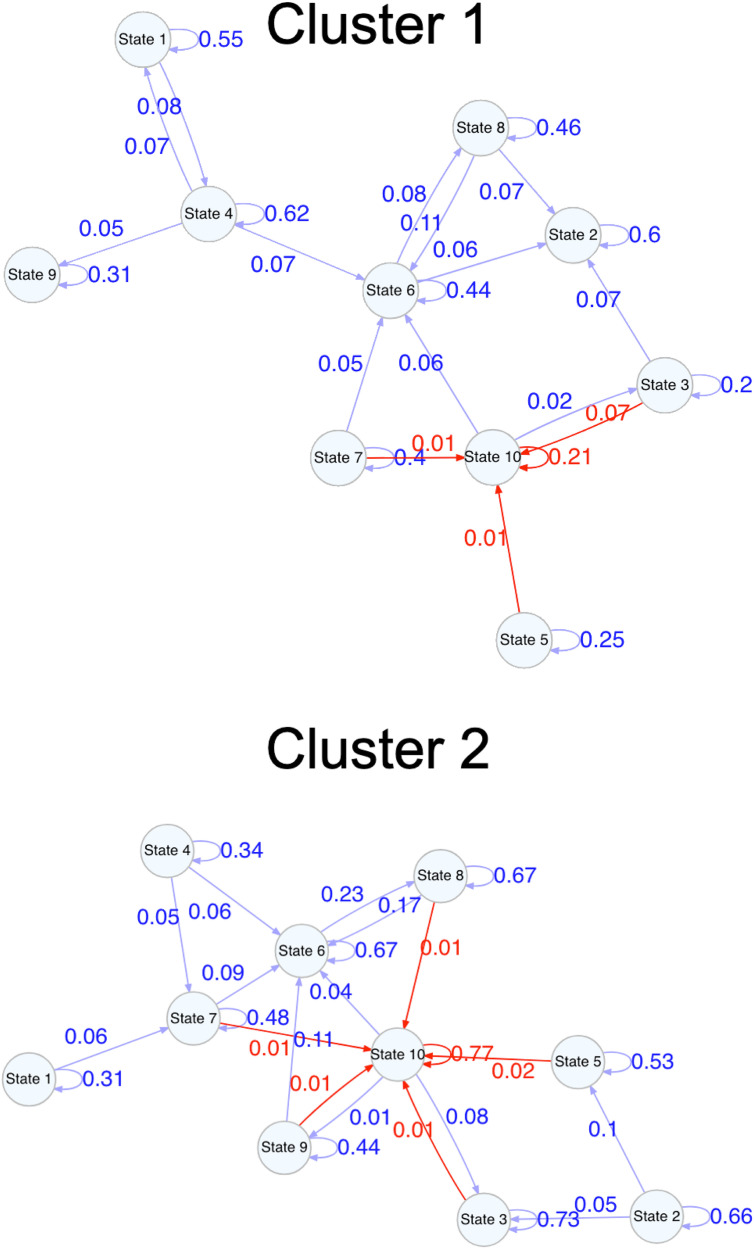
Transition probabilities between hidden states for clusters 1 and 2 in time segmentation 2. In this figure, when the transition probability is more than or equal to 0.05 (0.01 when state 10 is involved in the transition since state 10 is the most important state in this analysis), the transition probability and its path are denoted. The red color shows the transition where state 10 is involved.

Fig 7 shows the visualized transition probabilities for clusters 1 and 2. The transition patterns differ between the two clusters at some points. First, compared with cluster 1, state 10 tends to remain in cluster 2. This implies that, in cluster 2, once a customer enters a state of interest, such a state would continue for a while. By contrast, in cluster 1, this state of interest does not persist and is likely to change. Second, there are more paths to state 10 in cluster 2. In cluster 2, five states (states 3, 5, 7, 8, and 9) change to state 10 with more than or equal to 0.01. In contrast, in cluster 1, there are three paths (states 3, 5, and 7). Furthermore, in cluster 2, several paths change back to state 10. That is, when state 10 changes to another state, it may return to state 10 with some possibility. For illustration, State 10-State6-State8-State10 is a possibility. However, this is not the case for cluster 1. When state 10 changes to another state, this possibility is highly limited in cluster 1.

In summary, we determined how the occurrence pattern of cluster 2 in state 10 (i.e., the cluster that predicted successful travel consultations) was generated by closely examining the transition patterns of hidden states in time segmentation 2. We found that cluster 2 tended to stay in state 10 and that there were various patterns that returned to state 10, even when state 10 changed to another state.

## Discussion

### Contributions of the present study

In this study, we analyzed the interactions between clerks and customers during a 30-minute travel consultation. In particular, we analyzed how the interactions differed between successful and unsuccessful travel consultations. Our findings can be summarized as follows.

First, we performed analyses using the HMM and attempted to identify behavioral patterns that would separate successful from unsuccessful consultations. We found behavioral patterns that could predict successful travel consultations. Specifically, the occurrence of a particular state in the first 10 minutes of a 30-minute consultation was a strong predictor of its success. Previous studies have shown a relationship between the internal state and patterns of behaviors [7–12]. However, it may be difficult to use such findings to predict a successful 30-minute consultation. In particular, patterns of multiple behaviors were highly complicated because the experimental setting was as close as possible to a realistic travel consultation. Thus, the present findings can be regarded as novel since we identified the features that characterized the differences in interactive behavioral patterns between successful and unsuccessful consultations in a highly complicated real-world situation. Second, our findings provide new insights into how people evaluate events that last for a certain amount of time. Previous studies have shown that the evaluation process for events lasting for a certain amount of time can be explained by models such as the peak-end rule [23–25]. The present findings (i.e., the overall evaluation of the consultation was shaped early in the consultation process) might be inconsistent with the peak-end rule. However, our findings do not necessarily contradict those of previous studies. We note that the nature of the tasks was completely different. Previous studies did not examine events involving interactions. Thus, the present findings suggest that the evaluation process differs depending on whether an interaction is involved. In other words, we provide new evidence on how people evaluate events that involve interactions over time. In this sense, we can assume that the results of this study provide novel empirical findings in situations that have not been examined in previous studies, rather than contradicting the findings of previous studies.

The present study was designed to examine relatively short-term interactional dynamics within a single 30-minute consultation. The predictive value of State 10 reflects how early mutual engagement shapes the relatively short-term perception of consultation quality, rather than long-term relationship building or repeated interactions. This scope is central to the contribution of the research: by modeling moment-to-moment behavioral sequences in a naturalistic service setting, the study provides empirical evidence on how people evaluate unfolding interaction events over short time periods. Such evaluations are critical in many real-world service encounters—such as travel consultations or financial advising—where impressions are formed rapidly and often within a single session. By clarifying these short-term dynamics, the findings complement rather than replace research on longitudinal relationship formation, and they highlight the importance of early engagement behaviors in shaping immediate satisfaction outcomes.

Our results also resonate with prior service encounter research on the temporal dynamics of customer evaluations. In extended service encounters, early positive experiences tend to exert a disproportionately strong influence on overall satisfaction—a pattern consistent with the primacy effect [26]. Similarly, early rapport-building behaviors, such as attentive listening and personalized responses, serve as critical antecedents to trust and engagement in sales interactions [27]. The present finding that the early emergence of an interest-related, cooperative, and mutually focused state (State 10) predicts consultation success suggests that effective rapport formation at the beginning of the encounter establishes a positive relational frame, which facilitates smooth communication and alignment throughout the interaction.

We note that the present study does not aim to demonstrate the superiority of the HMM framework over simpler statistical approaches. Rather, the HMM is used as a descriptive tool to capture the temporal structure of interactional dynamics. In particular, it allows us to represent interactions as sequences of latent states defined by combinations of multiple behaviors and to examine how these states evolve over time, thereby providing a process-level perspective on interaction patterns that is difficult to obtain from aggregated or static measures alone. At the same time, the present findings should be interpreted primarily in the context of service interactions, rather than as direct evidence for general principles of evaluation of time-extended events. While previous research has examined how temporal structure influences retrospective evaluations, the current results offer a process-level perspective on how interactional dynamics unfold in a naturalistic setting. As such, the implications for broader theories of time-extended evaluation should be considered as suggestive rather than definitive, and future research will be necessary to examine whether similar patterns emerge in other contexts.

### Limitations and future directions

#### Data-related limitations.

Given the relatively small number of dyads, the present results should be interpreted as providing initial evidence of recurring interactional patterns observed in a naturalistic setting. While these findings offer insight into how such patterns may be associated with consultation outcomes, further research with larger and more diverse samples will be necessary to establish their generalizability and robustness.

Although the present study identified specific states that predict higher satisfaction, it should be noted that satisfaction ratings reflect subjective evaluations and may not necessarily correspond to actual behavioral outcomes. Previous meta-analytic evidence indicates that customer satisfaction is associated with, but distinct from, behavioral measures such as repurchase or loyalty [44]. In the current dataset, objective indices such as booking completion or follow-up contacts were not available, which limits our ability to assess behavioral consequences directly. Future research should therefore integrate post-session behavioral data—for example, whether a customer made a booking or revisited the agent—to examine whether the state-level patterns observed here predict concrete, economically relevant outcomes. Such integration would provide a more comprehensive validation of interactional success and further strengthen the ecological validity of this framework.

It should be noted that the present sample consisted exclusively of female clerks working at a travel agency in Japan. This sampling choice reflects the typical demographic composition of travel agency staff in the Japanese context and ensured ecological validity for the present study. However, it also limits the generalizability of the findings. In particular, backchannel behaviors such as nodding (“aizuchi”) and the timing of verbal responses are culturally embedded features of Japanese communication and may not have identical meanings or frequencies in other cultures. Similarly, gender-related communication styles—such as responsiveness or politeness strategies—could influence the dynamics observed in the current data. Future research should therefore aim to replicate these analyses with more gender-diverse and cross-cultural samples to determine whether the identified interactional patterns are universal or culture-specific.

Another limitation concerns potential confounding variables that may have influenced the interactional dynamics and consultation outcomes. Although some background information—such as customer demographics or characteristics of the proposed itinerary—was available in the dataset, the number of observations for these variables was insufficient to allow for reliable statistical control. For example, clerks and customers participated in multiple sessions, resulting in duplicate values for demographic variables such as age. Moreover, all clerks in the present study were female, making it impossible to examine or control for gender-related differences. Given these constraints, incorporating these variables into the statistical models would likely have produced unstable or uninterpretable estimates. Future research with larger and more balanced samples should therefore aim to collect such information to more clearly disentangle individual- and context-level influences on consultation success.

We divided each consultation into equal three-minute segments to examine how local interactional states unfolded over the course of the consultation. This segmentation was not intended to imply that three minutes represents a natural psychological boundary. Rather, it was chosen as an intermediate temporal unit that balances two competing requirements: retaining sufficient temporal resolution to capture changes in interactional dynamics, while ensuring that each segment contains enough behavioral observations to estimate the occurrence of HMM states reliably. This choice is consistent with prior research treating service encounters as sequences of events [45] and showing that the temporal distribution of events contributes to post-encounter evaluations [46]. It is also compatible with the thin-slice literature [47,48], which demonstrates that brief samples of interpersonal behavior, often shorter than five minutes, can contain meaningful information about social outcomes. Furthermore, additional analyses using alternative temporal segmentations (e.g., 8 and 12 segments) recovered the same interactional state associated with successful consultations as in the original 10-segment division (i.e., only State 10 showed posterior probabilities of consultation success whose 95% HDI did not overlap with 0.5), suggesting that the main findings are not tied to a specific segmentation scheme but instead reflect a general pattern in the interaction dynamics. Results of the analyses using the 8- and 12-segment divisions are reported in the Supplementary Materials.

### Measurement and operationalization limitations

We recorded the interactions between clerks and customers using videos. This method has the limitation of a strict measurement of behavior. To more strictly measure behaviors pertaining to interactions, participants may be required to wear devices for the measurements. However, there is a tradeoff between keeping the situation as close as possible to a realistic one and strictly performing the measurement. Future research should conduct new experiments with more emphasis on measurements and examine the validity of the present findings. Second, we analyzed the interaction between clerks and customers based on the behavioral patterns observed every second. Previous studies have shown that people can infer causal relationships between behaviors from sequences of behaviors [49–53]. In these studies, the researchers conducted relatively simple laboratory experiments. Thus, although these previous findings may not be directly applicable to the present issue (i.e., we examined complicated interactions in a real-world setting), we can take advantage of some of their insights. For example, during a travel consultation, a clerk and customer may have found some meaning in the sequence of the other’s actions, which may have influenced the interaction. In this study, we provided empirical findings on the nature of dyadic interactions using the HMM. In particular, we demonstrated differences in the interaction between successful and unsuccessful pairs. However, this study did not examine clerks’ and customers’ feelings toward the other’s behavioral patterns and how this was reflected in the interaction. Analyses of which causal relationships are inferred based on the other’s behavioral sequence will provide a variety of insights to clarify this point.

Although dynamic synchrony measures—such as motion energy analysis or cross-correlational attention synchrony—would provide a stronger test of interpersonal coordination, the present dataset did not contain sufficiently detailed motion information to compute such metrics. Previous studies have shown that fine-grained behavioral synchrony, including moment-to-moment coordination of movement, is a robust predictor of relationship quality and dyadic outcomes [21]. However, because the video data in the present study did not consistently capture participants’ full body movements, we were unable to apply such methods. To partially address this limitation, we examined static joint-attention indices derived from the available behavioral labels. Specifically, we calculated the proportion of time during which the customer was looking at the brochure (or at the clerk) and, at the same moment, the clerk was also looking at the same target (i.e., the brochure or the customer). In other words, we quantified the percentage of time during which both participants’ gaze behaviors were aligned toward the same object or person. The results of this analysis are presented in Table 5. These indices were higher in successful consultations than in unsuccessful ones. These findings are consistent with theories of joint attention and nonverbal immediacy, which propose that shared attentional focus and immediacy cues enhance interpersonal involvement and relational outcomes [54]. At the same time, these findings highlight the need for future research to incorporate dynamic synchrony measures—including motion energy analysis and cross-correlational time-series methods—to more precisely capture the moment-to-moment coordination of attention and bodily movement. Such analyses would enable a more comprehensive understanding of how nonverbal synchrony, joint attention, and immediacy behaviors contribute to successful service encounters.

**Table 5:** Proportion of time spent in static joint attention (simultaneous gaze toward the same target) and results of the t-test.

Descriptive statistics (mean and standard deviation)		
Pair	Brochure	Looking at each other
Successful	0.461(0.021)	0.269(0.076)
Unsuccessful	0.399(0.125)	0.231(0.115)
Results of t-test		
Brochure	*t*(28) = 1.969, *p* = 0.059, *d* = 0.721	
Looking at each other	*t*(28) = 1.078, *p* = 0.290, *d* = 0.394	

The present study identified distinct behavioral states using a data-driven approach, without assigning predefined conceptual labels to each hidden state. It is important to emphasize that the hidden states identified by the HMM are statistical constructs derived from observed behavioral patterns. As such, interpretations of these states in terms of psychological constructs—such as customer interest—should be regarded as inferential rather than definitive. In the present case, the interpretation of this state is based on a coherent and internally consistent pattern of behavioral indicators (see Fig 6), suggesting a meaningful interactional profile rather than an arbitrary statistical grouping. However, this pattern does not constitute direct evidence of an underlying psychological state and should instead be understood as being compatible with an engagement-related interpretation. Thus, although State 10 may be suggestive of engagement-related tendencies (e.g., customer interest), such interpretive labeling requires careful validation grounded in both empirical evidence and theoretical frameworks, such as nonverbal immediacy theory [55,56]. Future research combining behavioral data with independent measures of psychological states (e.g., self-reports) will be necessary to more rigorously examine the psychological meaning of these patterns. More broadly, future work should aim to investigate the causal and psychological mechanisms underlying each hidden state and to develop conceptually grounded labels that capture their behavioral and affective significance.

### Modeling limitations

In the present study, we adopted a conventional HMM with the number of states determined by BIC. This approach was chosen to align with the exploratory and empirical aims of the study. Alternative frameworks such as Hidden Semi-Markov Models (HSMMs) [57], or Sticky Hierarchical Dirichlet Process HMMs [58] offer valuable extensions, applying them would substantially expand the scope of the current work. At the same time, implementing HSMMs or HDP-HMMs in a principled way would require a substantial re-specification of the modeling framework, including additional modeling choices, hyperparameter tuning, and extensive validation (e.g., simulation studies, convergence checks, and sensitivity analyses). Given the size and structure of our current dataset, and the empirical focus of this paper on identifying interpretable behavior patterns linked to consultation success in a naturalistic field setting, we believe that such a comprehensive methodological extension would go beyond the intended scope of the present study.

Importantly, our sensitivity analyses with different state numbers and random seeds consistently recovered a state closely matching State 10, suggesting that the main conclusions are robust within the modeling framework adopted here. Future research—particularly studies with larger datasets and a methodological focus—should explore HSMMs and HDP-HMMs to more comprehensively model heterogeneous state durations and nonparametric state structures in service-interaction dynamics.

Notably, similar interactional patterns were observed across alternative model specifications with different numbers of hidden states. This suggests that the key findings of the present study do not depend on a specific choice of model complexity, but instead reflect more general features of the observed interaction dynamics. At the same time, given the relatively small size of the dataset, these results should be interpreted with appropriate caution, and further research will be necessary to examine the stability of these patterns in larger samples.

### Interdependence and individual-difference limitations

Interdependence and individual-difference limitation concern the potential nonindependence of the consultation data. Although each consultation was treated as an independent observation, it is possible that clerk-specific behavioral styles influenced the observed interactional patterns. A theoretically promising approach to address such interdependence is the Actor–Partner Interdependence Model (APIM [59]), which allows for the simultaneous estimation of actor effects (the influence of one’s own characteristics) and partner effects (the influence of the counterpart’s characteristics). However, the reliable estimation of actor and partner effects requires sufficient variability and sample size at both the individual and dyadic levels. In the present field-experimental dataset, each clerk interacted with only a few customers (three pairs on average), making it statistically difficult to obtain stable parameter estimates. Moreover, implementing the APIM would require additional covariates—such as personality traits or communication styles of the participants—to properly model individual-level differences. Because such detailed individual-difference measures were not collected in the current field setting, this type of analysis was not feasible. For these reasons, we focused instead on identifying the descriptive and predictive patterns of the interactional states. Future studies that involve larger samples and include relevant individual-difference variables will allow for a more rigorous examination of actor–partner interdependence while maintaining ecological validity.

## Conclusion

In conclusion, we demonstrated the characteristics of successful interactions in a 30-minute travel consultation. We found that successful consultations were characterized by the early emergence and persistence of a behavioral state compatible with customer engagement or interest. We believe that the present findings provide new evidence about dyadic interactions and make substantial contributions from a novel perspective toward understanding human-human interaction.

## Supporting information

S1 Appendix Supplementary analyses and methodological details. Contains additional information on questionnaire contents, annotation procedures, HMM analyses, annotation reliability, robustness checks, and supplementary temporal segmentation analyses.
(DOCX)
