# Supplementary material for: The occurrence of a particular state is a predictor of successful travel consultation
Source: PLoS One. 2026 Jun 22;21(6):e0352101. doi: 10.1371/journal.pone.0352101 (PMC13286169; doi:10.1371/journal.pone.0352101)
Supplement: S1 Appendix — Contains additional information on questionnaire contents, annotation procedures, HMM analyses, annotation reliability, robustness checks, and supplementary temporal segmentation analyses. (DOCX) [file pone.0352101.s001.docx]

**Supplementary Material**

Data can be accessed at the following URL.

<https://osf.io/qj5wv/>

**Question contents**

In addition to the question reported in the main text, clerks and customers were asked to answer the following questions.

Clerks

For each of the three to five specific travel plans, including hotels, sightseeing content, flights, etc., which the clerk proposed to the customer during the travel consultation, the clerks were asked to answer how attractive the customer thought the proposed plans were. For this question, the clerks answered using a seven-point scale (1: not at all attractive to 7: very attractive) .

Customers

For each of the three to five specific travel plans, including hotels, sightseeing content, flights, etc., which the clerk proposed to the customer during the consultation, the customers were asked to answer how attractive the proposed plans were. For this question, the customers answered using a seven-point scale (1: not at all attractive to 7: very attractive) .

These ratings were not used in the present analysis. In Honda et al. (2016), we analyzed these ratings and reported the results.

**Distribution of rating for travel consultation by clerks and customers**

Sup. Figure 1 shows the distribution of rating for trave consultation by clerks and customers. A wide range of ratings (from 1 to 7) were observed from clerks. In contrast, customers basically gave high ratings (higher than or equal to 4). Based on these rating characteristics, we set different criteria for clerks and customers with respect to success of consultation.


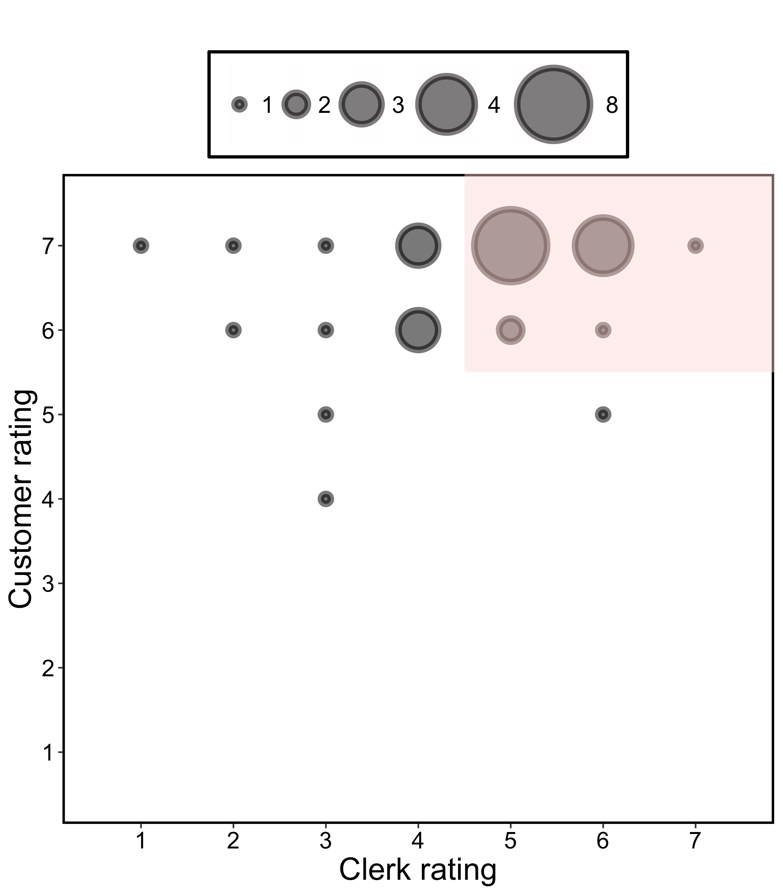


Sup. Figure 1. Distribution of rating for travel consultation. The bubble size demonstrates observed frequencies of ratings. The red shadow area in the figure indicates the “Successful pair.”

**Tagging rules for verbal and nonverbal behavior**

Nonverbal behaviors were annotated under the following rules.

| Lean forward | From start to finish when the upper body is considered to be contained in front of the desk. |
| --- | --- |
| Gaze | We considered three categories of direction of gaze: partner (i.e., clerk to customer, or customer to clerk), brochure, and anything not included in these two. Under this categorization, we annotated the gaze that directed toward the partner and toward the brochure. |
| Nod | One nod was converted as appearing for one second. |
| Chin on the hand | From start to finish with hand on chin |

**Hidden Markov Model (HMM) used for the present analyses**

We analyzed the present data using HMM. We used “hmm.discnp” library in R (Turner, 2020). This library can be applied for the discrete observation data. In the present study, we regarded the observed behavior patterns [i.e., the combination of the four clerks' and six customers' behaviors, see Table 1 in the main text] as discrete observations.

As described in the main text, there are 2^10^(= 1024) different behavior patterns at most in possibility. However, we observed 416 different behavior patterns in the whole 30 pairs. Thus, for each hidden state, emission probability for each of the 416 patterns was calculated.

**Analyses of annotation validity**

The recorded movies were annotated by the third author (R.H), and we used these annotated data for the present analyses. We checked validity of the annotation in terms of the following consistency and robustness.

1: Consistency

First, we examined the annotations in terms of consistency between independent raters. For this, 10 videos were randomly selected, and then the first 10 minutes of those videos were annotated. These ten videos were annotated by in total seven raters. Four of raters annotated one video, and the other three raters annotated different two videos. These seven raters were not informed of the purpose or hypotheses of the present study, and the rules for annotation were exactly the same as those when the third author annotated the videos.

We then calculated the consistency rate of the annotations between two independent raters. In calculating consistency rates, we checked whether the annotation was consistent for each second. Descriptive statistics of the agreement rates are as follows.

| Mean | Median | SD | Range |
| --- | --- | --- | --- |
| 0.832 | 0.831 | 0.045 | 0.758-0.889 |

Given that we checked the consistency of annotations for each second, the consistency rates would be high.

2: Robustness of analyses using HMM

The consistency rate of annotations between two independent raters seems high as we described above. However, it is not obvious how the differences in annotations affect the results of the analyses. Therefore, the following analysis was conducted to examine how the differences in annotations affect the results of the analysis in terms of the robustness.

Using the HMM results in the main text, we analyzed how the estimated hidden state changes with different annotation data. Here, we calculated consistency of estimated hidden states when the data of the first 10 minutes replaced with each other. Then, we divided the travel consultation into 10 segments as in the analysis in the main text, and then calculated the consistency of occurrence rate of each hidden state in each time segment. We used correlation coefficient as the index of consistency. We found that the mean (median) of the correlation coefficient between the different data for the 10 segments was 0.832 (0.860).

Based on the results of the analyses in terms of consistency and robustness, we conclude that the annotated data used in the main text is basically consistent regardless of raters, and that differences in annotations depending on different raters do not critically affect the results of the analysis.

**Effects of time window selection on cross-validated AUC estimates**

This analysis examines the stability of cross-validated AUC estimates obtained using different time windows and segment combinations. The aim of this analysis is to clarify how restricting the observation window influences the reliability and discriminative power of AUC-based predictive validation under the cross-validation procedure used in the main analysis.

To this end, we conducted additional predictive validation analyses using narrower time windows than the 6–15 min window reported in the main text. Specifically, we examined (i) the 6–9 min window (corresponding to time segment 3 only; n = 30) and (ii) the 6–12 min window (time segments 3 and 4; n = 60). For each setting, we applied the same repeated 10-fold cross-validation procedure as in the main analysis and computed the area under the ROC curve (AUC) as the performance metric.

When the analysis was restricted to the 6–9 min window (N = 30), the estimated AUC values were highly unstable across cross-validation repetitions. Notably, all hidden states exhibited AUC values exceeding 0.85, resulting in uniformly high performance estimates with little meaningful differentiation between states. This pattern indicates that, under this restricted setting, AUC estimates were dominated by variability due to random data partitioning rather than reflecting genuine predictive differences among states.


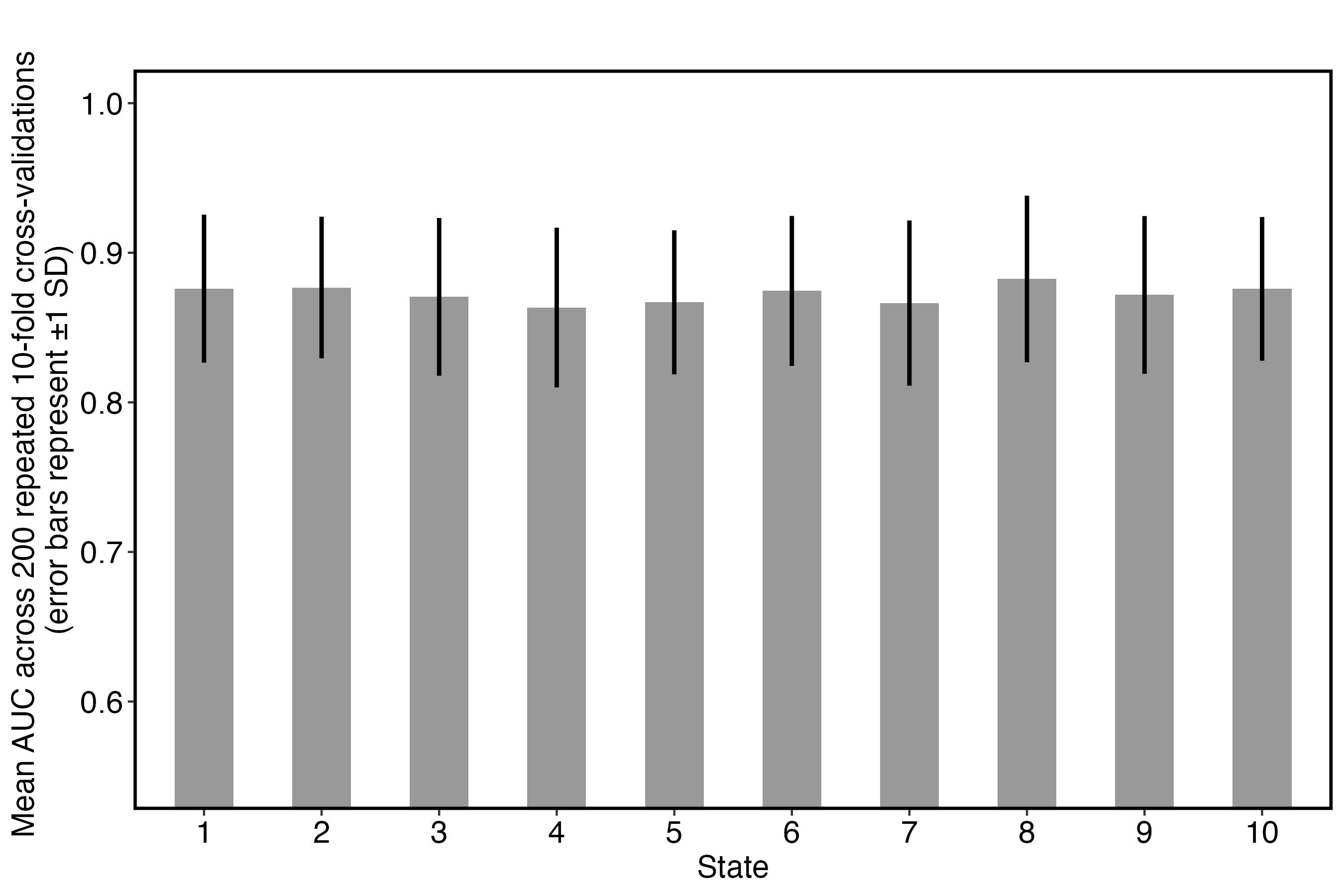


When the analysis was extended to the 6–12 min window (N = 60), some differentiation between states began to emerge; however, multiple states still exceeded an AUC of 0.7, and the overall pattern remained insufficiently selective. Thus, although increasing the sample size partially reduced instability, cross-validation–based performance estimates remained sensitive to random splits and did not reliably distinguish theoretically relevant states from others.


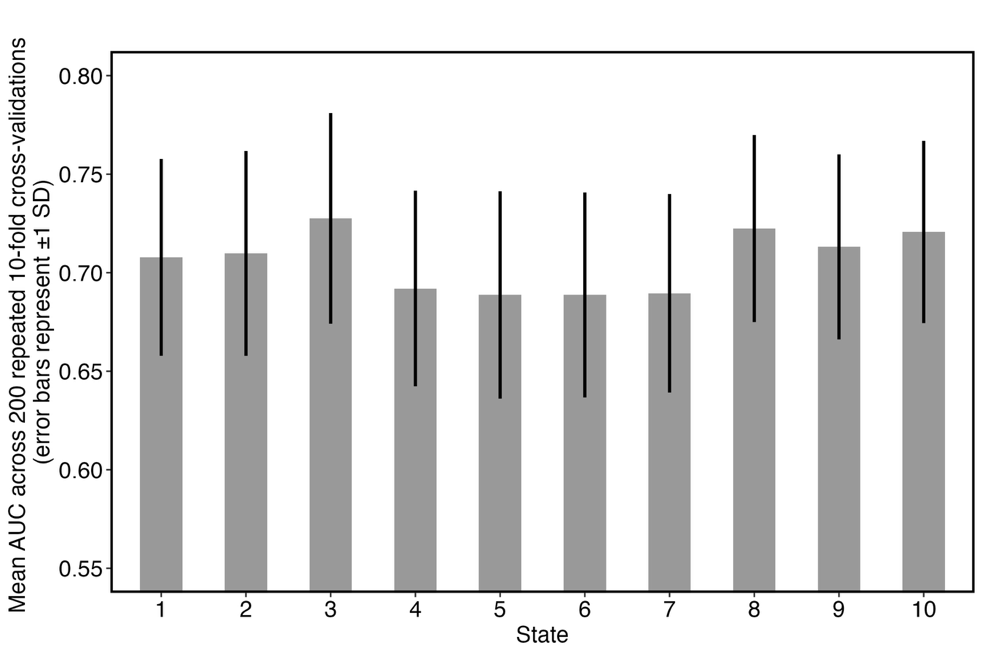


In contrast, using the full 6–15 min window (N = 90) yielded a qualitatively different pattern, as reported in the main text. Under this setting, only State 10 consistently achieved an AUC above 0.7 (AUC = 0.712), whereas all other states remained below this threshold, resulting in stable and discriminative performance estimates across repetitions.

These findings are consistent with prior methodological work demonstrating that, in small-sample regimes, cross-validation results can be highly variable and strongly dependent on random data splits, thereby hindering reliable model comparison (de Rooij & Weeda, 2020). Moreover, resampling-based estimates of predictive performance have been shown to become overly optimistic when sample sizes are small, particularly when non-linear evaluation metrics such as AUC are used (Molinaro et al., 2005). In such settings, uniformly high AUC values across models are more indicative of evaluation instability than of genuine predictive accuracy.

Taken together, these supplementary analyses support the choice of the 6–15 min window in the main analysis. This window represents the smallest setting in which cross-validation yielded stable and discriminative AUC estimates, allowing meaningful comparison across hidden states while avoiding the instability observed under more restrictive data conditions.

**Results of analyses using different time segments.**

The figures below show the mean emission probabilities for the two clusters as a function of temporal division. Error bars denote standard errors.


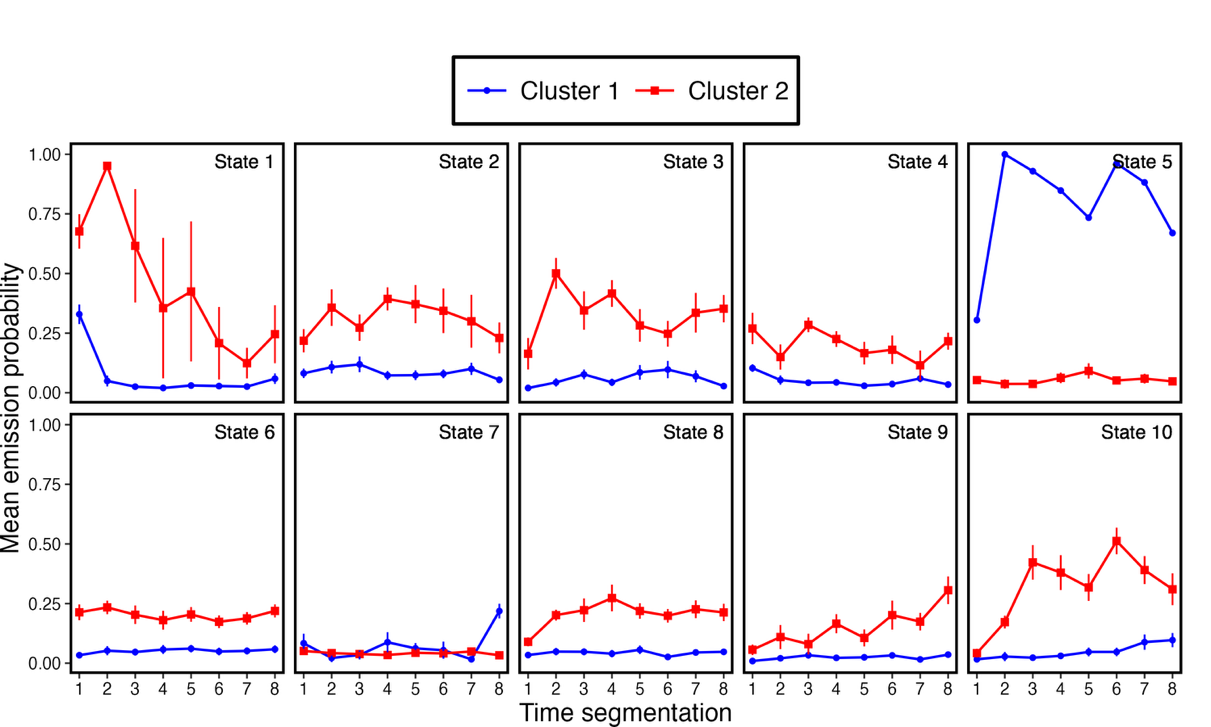


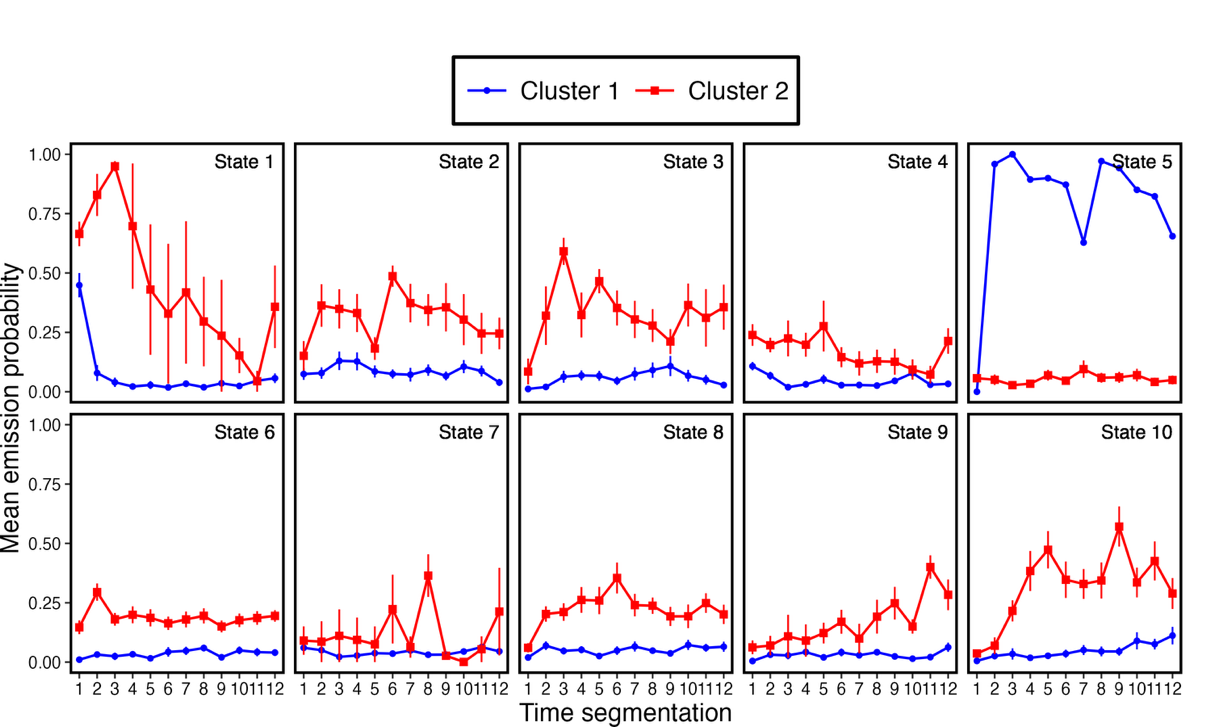


The figure below shows the posterior probability of a successful pair given the observed emission pattern. Points denote the medians of the posterior distributions, and error bars represent the 95% highest density intervals.


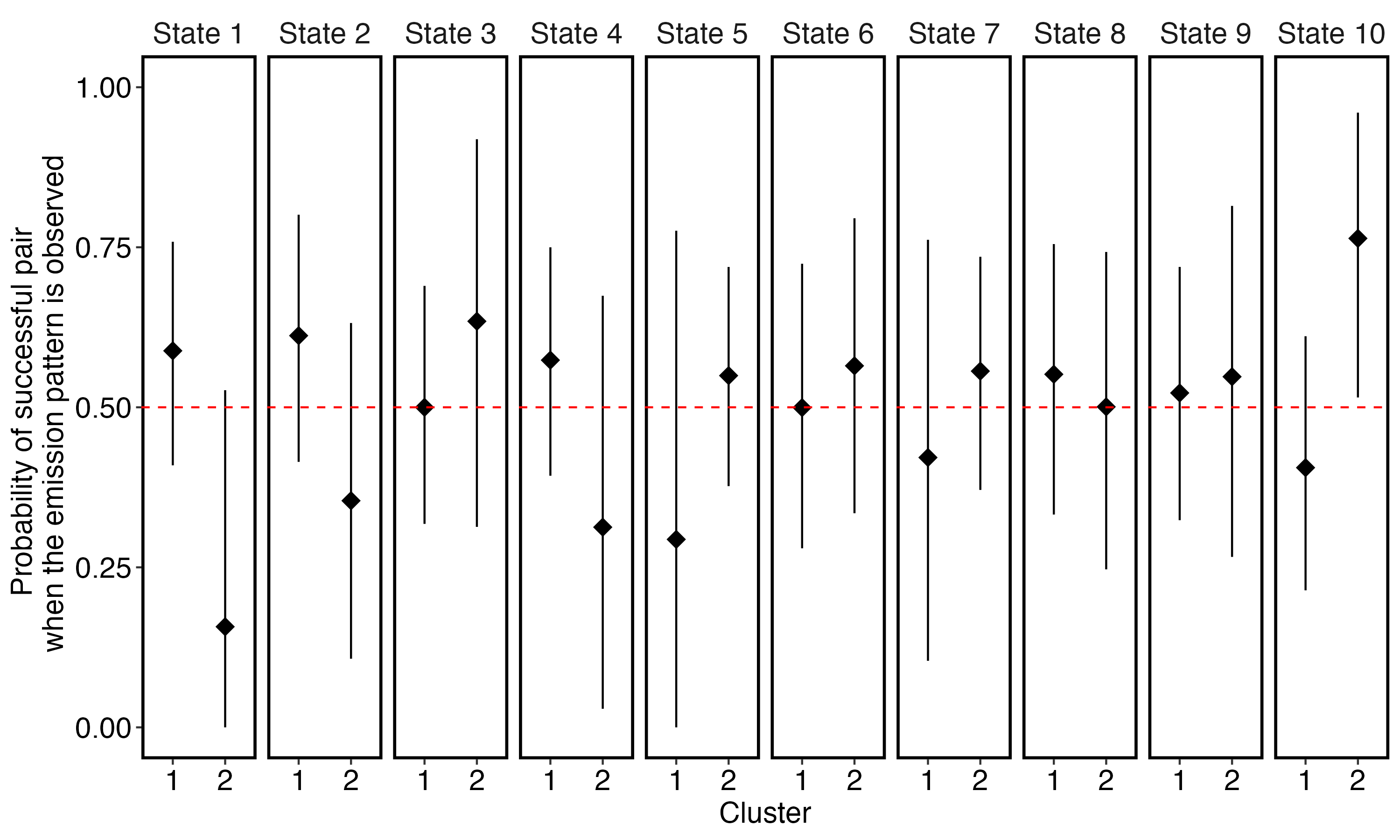


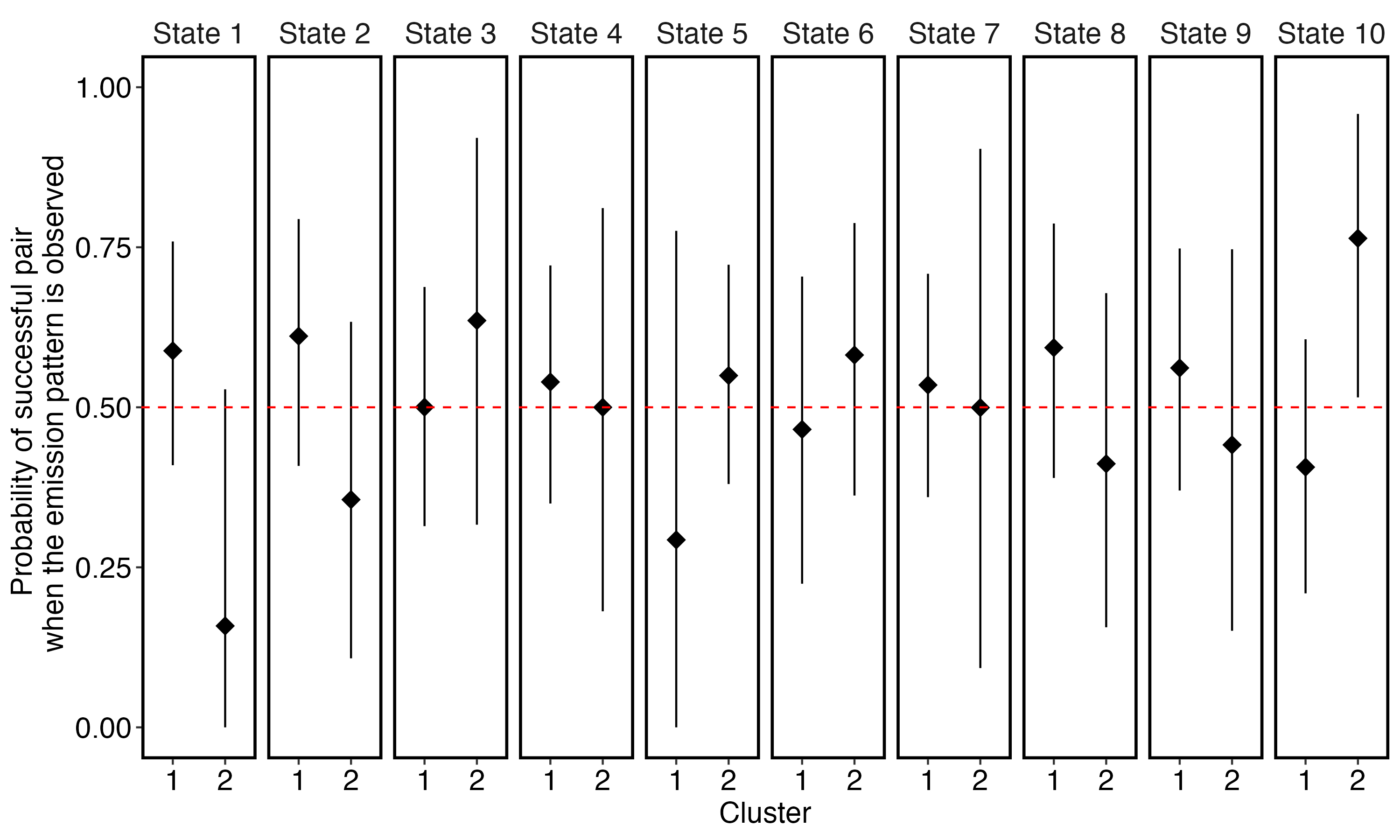


**References**

de Rooij, M., & Weeda, W. (2020). Cross-validation: A method every psychologist should know. Advances in Methods and Practices in Psychological Science, 3(2), 248–263.

Honda, H., Hisamatsu, R., Ohmoto, Y., & Ueda, K. (2016). Interaction in a natural environment: Estimation of customer’s preference based on nonverbal behaviors. *Proceedings of the Fourth International Conference on Human Agent Interaction* (HAI ’16), 93–96.

Molinaro, A. M., Simon, R., & Pfeiffer, R. M. (2005). Prediction error estimation: a comparison of resampling methods. Bioinformatics, 21(15), 3301–3307.

Turner, R. (2020). hmm.discnp: Hidden Markov models with discrete non-parametric observation distributions. R Package, Version 3.0-6. https://cran.r-project.org/web/packages/hmm.discnp/index.html
